# Development and Clinical Validation of an Automated LLM Judge for Evaluating Perioperative Patient Questions

**DOI:** 10.3390/bioengineering13101115

**Published:** 2026-09-24

**Authors:** Bernardo G. Collaco, Nadia G. Wood, Yunguo Yu, Carina Rosa Malena, Srinivasagam Prabha, Ashton L. Boon, Zhihui Fang, Anjali Bhagra, Bradley C. Leibovich, Antonio Jorge Forte

**Affiliations:** 1 Division of Plastic Surgery, Mayo Clinic, 4500 San Pablo Rd S, Jacksonville, FL 32224, USA; 2 Department of Radiology AI IT, Mayo Clinic, Rochester, MN 55905, USA; 3 Center for Digital Health, Mayo Clinic, Rochester, MN 55905, USA; 4 Department of Administration, Mayo Clinic, Rochester, MN 55905, USA; 5 Division of Clinical Trials and Biostatistics, Mayo Clinic, Jacksonville, FL 32224, USA; fang.zhihui@mayo.edu; 6 Department of Medicine, Mayo Clinic, Rochester, MN 55905, USA; 7 Department of Urology, Mayo Clinic, Rochester, MN 55905, USA; 8 Department of Artificial Intelligence and Informatics, Mayo Clinic, Jacksonville, FL 32224, USA

**Keywords:** large language models, LLM-as-a-judge, artificial intelligence, clinical validation, perioperative care

## Abstract

Background: Large language models (LLMs) are increasingly used in patient-facing clinical applications, creating a need for scalable methods to evaluate the accuracy, safety, and appropriateness of their responses. Although physician review remains the reference standard, it is resource-intensive and difficult to scale. Objective: To develop and clinically validate an LLM-as-a-judge framework for evaluating responses to patients’ periprocedural questions. Methods: We retrospectively evaluated 1285 patient–AIVA interactions from 112 patients across two Mayo Clinic sites and multiple surgical specialties. Physician reviewers classified interactions using a predefined true-positive (TP), false-negative (FN), true-negative (TN), and false-positive (FP) framework. The finalized LLM judge independently evaluated the same interactions. Agreement was assessed using four-class and category-specific agreement, Cohen’s kappa, and a secondary binary analysis of response correctness. Results: Overall, four-class agreement was 93.1% (95% CI, 91.7–94.5%), with Cohen’s κ = 0.852 (95% CI, 0.823–0.882). Category-specific agreement was 94.1% for TP, 90.7% for FN, 95.5% for TN, and 84.9% for FP classifications. In the binary correctness analysis, accuracy was 93.4% (95% CI, 92.0–94.7%), sensitivity 94.6%, specificity 90.2%, precision 96.2%, and F1-score 0.954. McNemar’s test demonstrated no significant asymmetry between paired classifications (*p* = 0.11). Conclusions: A clinically grounded LLM judge closely approximated physician evaluation of patient-facing AI responses. These findings support its potential as a scalable assistive monitoring tool while preserving physician oversight for uncertain or safety-sensitive interactions.

## 1. Introduction

Large language models (LLMs) are increasingly being used to generate responses to patients’ questions, including questions related to preparation for, recovery from, and expectations surrounding medical procedures [1,2,3]. As these tools become more widely incorporated into patient-facing applications, rigorous evaluation of the quality, accuracy, and safety of their responses is essential [4]. Traditionally, such assessments rely on review by clinicians or other subject-matter experts. Although expert evaluation remains the reference standard, it is time-intensive, costly, and difficult to scale to the large volumes of content that can be generated by LLMs [5].

The use of an LLM as an automated evaluator, commonly referred to as an “LLM-as-a-Judge” approach, has emerged as a potential strategy for increasing the efficiency and reproducibility of response assessment [1,6]. In this framework, an LLM applies predefined evaluation criteria to score or compare generated answers, potentially enabling rapid screening of large numbers of responses before or alongside expert review (Figure 1). Prior work has demonstrated substantial agreement between capable LLM judges and human evaluators in general-domain language-model evaluation [6]. However, the validity of this approach in clinically relevant settings remains uncertain. LLM-based judges may exhibit systematic biases, including preferences for longer or more stylistically polished responses, sensitivity to prompt formulation, variability across repeated evaluations, self-preference when assessing outputs generated by the same or related models, and difficulty detecting subtle but clinically meaningful inaccuracies [1,7,8].

These limitations are concerning when evaluating responses to patients’ procedural questions. Guidance regarding medication management, dietary restrictions, procedural preparation, expected symptoms, and postoperative care can directly influence patient behavior, and incomplete, misleading, or unsafe information may have important clinical consequences [3,9]. Accordingly, an automated evaluator intended for this setting should demonstrate adequate agreement with expert clinical judgment and reliably distinguish high-quality responses from those containing clinically relevant deficiencies [10].

The objective of this study was to validate an LLM-as-a-Judge framework for evaluating answers to patients’ periprocedural questions by comparing LLM-generated assessments with expert human evaluations. We sought to determine whether an LLM Judge could provide a reliable and scalable method for assessing the quality and safety of patient-facing responses and thereby serve as a potential screening or complementary tool to expert review.

## 2. Materials and Methods

(a) Study Design

We conducted a retrospective methodological validation study evaluating a large language model (LLM) as an automated judge of responses generated by an artificial intelligence virtual assistant (AIVA). The study was nested within an ongoing investigation of AIVA for answering patient questions related to periprocedural care, approved by the Mayo Clinic Institutional Review Board (IRB #25-010999).

AIVA is a patient-facing conversational system using gemini-2.5-flash (gemini-2.5-flash; Google LLC, Mountain View, CA, USA), developed and initially tested using simulated postoperative patient questions [11], designed to answer periprocedural questions using approved clinical educational content and to provide appropriate escalation or redirection when questions cannot be safely addressed within its intended scope.

The study included 112 patients from Mayo Clinic sites in Rochester, Minnesota, and Jacksonville, Florida, across multiple surgical specialties, including plastic surgery, orthopedic surgery, oncologic reconstruction, and neurosurgery. Patients generated 1285 interactions with AIVA between 5 March and 6 August 2026 (Supplementary File S1). Patients could contribute more than one interaction, and the individual patient–AIVA interaction was the primary unit of analysis. Each interaction included the patient question, relevant clinical and procedural context, and AIVA response.

Before application of the LLM Judge, each interaction was independently evaluated by two physicians from a pool of three physician reviewers, including a board-certified plastic surgeon, using a predefined classification framework. The two primary reviewers were blinded to each other’s assessments. When disagreement occurred, the case was discussed and adjudicated, with involvement of the third physician reviewer when necessary, and the resulting consensus classification served as the final physician reference standard. These physician classifications served as the reference standard for assessment of AIVA performance, in accordance with established Human–LLM evaluation approaches [5]. Pre-adjudication reviewer classifications and discordant cases are provided in Supplementary File S2, and the interrater agreement analysis is provided in Supplementary File S3.

The LLM judge subsequently evaluated the same interactions independently and retrospectively. Judge-derived classifications were compared with the physician reference standard to determine whether automated evaluation could reproduce human assessment of AIVA performance. The judge did not influence AIVA responses or patient care (Figure 2).

(b) Scope Definition and Classification

AIVA’s scope was defined according to its intended function within the parent study (Figure 3). In-scope questions included patient inquiries related to periprocedural care before, during, or after a procedure, including procedural preparation, fasting, medication management, expected symptoms, recovery, activity restrictions, potential complications, indications for contacting the clinical team, and emergency escalation for predefined red-flag symptoms.

An in-scope interaction was classified as a true positive (TP) when AIVA provided an accurate, relevant, and safe response supported by approved Health Education Content (HEC) or appropriately escalated a predefined emergency scenario. An in-scope interaction was classified as a false negative (FN) when the response was incorrect, incomplete, inappropriate, unavailable because of a retrieval or knowledge gap, or failed to provide appropriate escalation. Unnecessary emergency escalation for a nonemergent concern was also classified as FN.

Out-of-scope (OOS) interactions included content outside AIVA’s approved clinical and operational functions, such as standalone acknowledgments, administrative requests, cost or insurance questions, unsupported access-related questions, questions about AIVA itself, staff feedback, expressions of gratitude, and conversational filler.

An OOS interaction was classified as a true negative (TN) when AIVA appropriately declined, redirected, or otherwise managed the interaction without unsupported engagement. An OOS interaction was classified as a false positive (FP) when AIVA inappropriately engaged with the interaction, including by providing substantive information outside its approved use case or available evidence.

(c) LLM-as-a-Judge Construction

Model selection and configuration

The LLM-as-a-Judge classifier was implemented as a one-call LLM Judge using gemini-2.5-flash through Vertex AI. For all validation classifications, the temperature was set to 0.0, the maximum output length to 8192 tokens, and the thinking budget to 1024 tokens. Up to three attempts were permitted in the event of a failed classification request. The model was accessed on 30 June 2026. These parameters were held constant throughout evaluation of the validation cohort.

For each patient–AIVA interaction, the selected model received a single structured prompt containing the patient question, AIVA response, surgical procedure context, specialty when available, and approved guideline evidence. The prompt operationalized the physician review rubric as a sequential decision tree: first determining whether the question was within AIVA’s procedure scope, then evaluating answer quality for in-scope questions or whether AIVA appropriately declined OOS questions, and finally assigning the TP, FN, TN, or FP label.

The classifier provided a structured JSON response that included the in-scope determination and rationale, answer-quality category and rationale, guideline reference, safety-concern severity and detail, final classification, and classifier confidence. High, medium, and low classifier confidence values were mapped to numeric confidence values for downstream reporting.

Clinical evidence and knowledge base

For each interaction, the LLM-as-a-Judge classifier received the evidence excerpts retrieved during the original AIVA interaction through the retrieval_context field. These interaction-specific excerpts, together with the patient question, AIVA response, procedure context, and specialty, were provided to the classifier as contextual evidence. Importantly, the Judge was not limited to the evidence retrieved by AIVA and also had access to an independent clinical knowledge base to support its evaluation and reasoning. This independent knowledge base included approved HIL/PEL content, medication-related evidence and context, and other approved clinical guidance.

Within the prompt, the collected chunks were rendered as guideline_evidence and treated as the primary evidence source for clinical correctness [12]. The Judge could therefore consider both the evidence associated with the original AIVA interaction and relevant information available through its independent knowledge base. The Judge was instructed to identify the evidence supporting its assessment whenever possible, enabling physician review of the rationale underlying TP, FN, TN, and FP classifications.

When no applicable guideline evidence was available, the prompt explicitly indicated the absence of supporting evidence. In such cases, the Judge could fall back on general procedural standards within the language model’s knowledge, while the guideline_reference field remained null. The structured output preserved the evidence associated with each classification as chunk_text, together with the Judge’s other classification outputs.

Prompt development and refinement

The judge prompt was designed to operationalize the physician review framework as a sequential classification task [6]. Explicit definitions were provided for in-scope and OOS interactions, response correctness, safety considerations, and the mapping of these determinations to TP, FN, TN, and FP classifications. Decision and tie-breaking rules addressed clinically relevant boundary cases, including emergency symptoms, wound and drain concerns, medication questions, cost or insurance inquiries, administrative requests, conversational filler, and questions unrelated to the patient’s procedure.

Few-shot examples were included to demonstrate representative classifications, including appropriate clinical guidance, clinically incorrect responses, inappropriate engagement with OOS questions, emergency escalation, appropriate refusal, and medication-related scenarios [13].

Before application to the validation cohort, two study physicians prospectively interacted with AIVA using 132 simulated patient-style questions representing in-scope, OOS, and clinically relevant edge cases (Supplementary File S4). The use of physician-generated questions was intentional and limited to prompt development and refinement, as this approach allowed the study team to deliberately probe predefined scenarios, establish the expected classification independently of the LLM judge, and efficiently identify ambiguous decision boundaries and recurrent classification errors [14]. These simulated interactions were not intended to replicate the full variability or natural language characteristics of real patient questions and were not used as a substitute for patient-generated data in the validation analysis.

The LLM Judge was subsequently applied retrospectively to this physician-annotated development set, and discrepancies between judge and physician classifications were reviewed to inform iterative refinement of the prompt and its tie-breaking rules. The finalized prompt was then applied without further modification to the real-world patient interaction cohort used for validation. The complete prompt, including definitions, decision rules, few-shot examples, and output schema, is provided in Supplementary File S5.

(d) Evaluation Tools

Physician-derived classifications served as the reference standard for evaluating AIVA. The LLM Judge independently classified the same interactions using the predefined TP, FN, TN, and FP framework.

Evaluation was performed at three levels. First, the original four-category classifications were used to calculate AIVA performance metrics independently from physician and LLM Judge assessments [11,15]. Accuracy, sensitivity (recall), specificity, positive predictive value (PPV; precision), negative predictive value (NPV), and F1-score were calculated as follows:

$$Accuracy=\frac{TP+TN}{TP++TN+FP+FN}$$

$$Sensitivity=\frac{TP}{TP+FN}$$

$$Specificity=\frac{TN}{TN+FP}$$

$$PPV=\frac{TP}{TP+FP}$$

$$NPV=\frac{TN}{TN+FN}$$

$$F1=2\times \frac{Precision\times Recall}{Precision+Recall}$$

Second, concordance between the LLM Judge and the physician reference standard was assessed using exact four-class agreement and category-specific agreement. Third, as a secondary analysis, the four original categories were collapsed according to response correctness: TP and TN were considered Correct, whereas FP and FN were considered Incorrect. This binary analysis evaluated the ability of the LLM Judge to reproduce the physician determination of whether an AIVA response was correct, irrespective of its original four-category classification.

Finally, to further characterize the predominant TP-to-FN disagreement pattern, a qualitative review was performed for interactions classified as TP by physician review but FN by the LLM Judge (Supplementary File S4). The patient question, AIVA response, supporting evidence, and Judge rationale were reviewed, and each interaction was assigned a primary descriptive theme representing the predominant reason for disagreement. This analysis was exploratory and specifically intended to characterize potential over-flagging by the LLM Judge rather than to provide a comprehensive qualitative analysis of all discordant classifications.

(e) Statistical analysis

Categorical data were summarized using frequencies and percentages. AIVA performance metrics were calculated independently using the TP, FN, TN, and FP classifications assigned by physicians and by the LLM Judge. Point estimates and 95% confidence intervals (CIs) were reported.

For the primary agreement analysis, a 4 × 4 matrix cross-classified the LLM Judge classification against the physician reference-standard classification. Overall agreement was defined as the proportion of interactions assigned the same category by both evaluators. Cohen’s kappa (κ) was used to quantify agreement beyond chance [16], and 95% CIs were calculated using the binomial method for overall agreement and the standard error method for κ. Category-specific agreement was calculated for TP, TN, FP, and FN as the proportion of interactions assigned to each category by the physician reference standard that received the same classification from the LLM judge; 95% CIs were calculated using the binomial method.

For the secondary binary analysis, Correct (TP or TN) was treated as the positive condition and Incorrect (FP or FN) as the negative condition, with physician evaluation as the reference standard. LLM judge performance was summarized using accuracy, sensitivity, specificity, PPV, NPV, and F1-score. Ninety-five percent CIs were calculated using the binomial method for accuracy, sensitivity, specificity, PPV, and NPV and the delta method for F1-score. Cohen’s κ assessed agreement beyond chance [16], and McNemar’s test evaluated asymmetry in paired binary classifications [17]. All tests were two-sided, with *p* < 0.05 considered statistically significant. Analyses were performed using SAS version 9.4 (SAS Institute Inc., Cary, NC, USA).

## 3. Results

(a) Study Cohort and Classification Distribution

The validation cohort included 1285 patient–AIVA interactions (Figure 4). Based on physician review, 884 interactions were classified as TP, 324 as FN, 44 as TN, and 33 as FP. The LLM Judge classified 862 interactions as TP, 344 as FN, 51 as TN, and 28 as FP (Table 1).

AIVA performance estimates derived from the LLM Judge were generally similar to those derived from physician evaluation (Table 2). Physician-derived accuracy was 72.2% (95% CI, 69.7–74.6%), compared with 71.1% (95% CI, 68.5–73.5%) using the LLM judge. Sensitivity was 73.2% versus 71.5%, and PPV was 96.4% versus 96.9%, respectively. F1-scores were also similar (0.832 vs. 0.823). The largest descriptive difference was observed for specificity, which was 57.1% with physician evaluation and 64.6% with the LLM judge; NPV was 12.0% and 12.9%, respectively.

Across metrics, absolute differences between physician- and Judge-derived estimates were small, ranging from 0.5 percentage points for PPV to 7.5 percentage points for specificity, with differences of 1.1 percentage points for accuracy and 1.7 percentage points for sensitivity. Overall, these findings indicate that use of the LLM Judge produced a broadly similar characterization of AIVA performance to that obtained from physician-derived classifications.

(b) LLM-as-a-Judge and Human Evaluation Agreement

The LLM judge and physician reference standard assigned the same TP, FN, TN, or FP classification in 1196 of 1285 interactions, corresponding to an overall agreement of 93.1% (95% CI, 91.7–94.5%). Cohen’s κ was 0.852 (95% CI, 0.823–0.882), indicating high agreement beyond chance (Table 3).

Agreement remained high across all four classification categories (Table 4). Category-specific agreement was highest for TN classifications at 95.5% (42/44; 95% CI, 89.3–100%) and TP classifications at 94.1% (832/884; 95% CI, 92.6–95.7%). Agreement was 90.7% for FN classifications (294/324; 95% CI, 87.6–93.9%) and 84.9% for FP classifications (28/33; 95% CI, 72.6–97.1%). The LLM Judge reproduced more than 90% of physician classifications for TP, TN, and FN interactions, whereas agreement was somewhat lower for FP interactions. The wider CIs for FP and TN agreement reflect the relatively small number of physician-classified FP interactions (n = 33), resulting in greater uncertainty around this category-specific estimate.

Among the 89 discordant classifications, the most frequent disagreement occurred when an interaction classified as TP by physicians was classified as FN by the LLM judge (n = 50). The second most frequent pattern occurred when physician-classified FN interactions were classified as TP by the LLM judge (n = 23). Less frequent disagreements included physician-classified FN responses assigned as TN (n = 7), FP responses assigned as TP (n = 5), TN responses assigned as TP (n = 2), and TP responses assigned as TN (n = 2).

(c) Binary Classification of Response Correctness

In the secondary binary analysis, 928 responses were classified as Correct and 357 as Incorrect by the physician reference standard. The LLM judge classified 913 responses as Correct and 372 as Incorrect. Of the physician-classified Correct responses, 878 were also classified as Correct by the LLM judge, whereas 50 were classified as Incorrect. Among physician-classified Incorrect responses, 322 were correctly identified as Incorrect and 35 were classified as Correct (Table 5).

Binary classification accuracy was 93.4% (95% CI, 92.0–94.7%), with sensitivity of 94.6% (95% CI, 93.2–96.1%) and specificity of 90.2% (95% CI, 87.1–93.3%). PPV was 96.2% (95% CI, 94.9–97.4%), NPV was 86.6% (95% CI, 83.1–90.0%), and the F1-score was 0.954 (95% CI, 0.944–0.964). Thus, agreement was somewhat higher for physician-classified Correct responses, of which 878/928 (94.6%) were also classified as Correct by the LLM Judge, than for physician-classified Incorrect responses, of which 322/357 (90.2%) were also classified as Incorrect. Among the 85 discordant binary classifications, 50 involved physician-classified Correct responses classified as Incorrect by the Judge, whereas 35 involved physician-classified Incorrect responses classified as Correct. Agreement beyond chance remained high, with κ = 0.837 (95% CI, 0.804–0.871). McNemar’s test showed no statistically significant asymmetry in paired classifications (*p* = 0.11).

(d) Qualitative Review of Discordant Cases

A descriptive review of the 50 interactions classified as TP by physicians but FN by the LLM Judge identified several recurrent patterns underlying disagreement (Table 6 and Supplementary File S6). The most common involved incomplete or insufficiently specific responses (25/50, 50%), in which AIVA provided clinically relevant information but did not directly address the specific timing, technique, prognosis, appearance, or rationale requested by the patient. Generic or overly conservative emergency escalation accounted for 10/50 cases (20%), particularly for postoperative symptoms in which the response directed patients to emergency services without addressing the specific concern or providing care-team escalation guidance. Other discordances involved context or evidence mismatch (7/50, 14%), temporal ambiguity or conflicting recovery guidance (5/50, 10%), and omission of safety or escalation information considered important by the Judge (3/50, 6%). Overall, these cases suggest that Judge over-flagging predominantly occurred when the Judge applied a stricter threshold for specificity, contextualization, or escalation guidance than the physician reviewer.

## 4. Discussion

(a) Clinical Implications

A distinguishing feature of this study is the validation of a clinically grounded Judge on real-world patient–AI interactions using the same structured classification framework applied by physician reviewers. Agreement between the LLM judge and the physician reference standard was 93.1%, with a Cohen’s κ of 0.852, indicating high agreement beyond chance. The high agreement across all four classification categories and the similar aggregate assessment between evaluation approaches suggest that, within a clearly defined evaluation framework, an LLM judge can approximate physician assessment without systematically producing more favorable or more unfavorable estimates of overall system performance.

These findings are consistent with emerging evidence that LLM-based evaluators can achieve substantial alignment with clinicians when the evaluation task is well structured and supported by explicit criteria. Croxford et al. (2025) validated an LLM-as-a-judge approach for evaluating clinical summaries and reported strong agreement with human evaluators [18], with the best-performing model achieving an intraclass correlation coefficient of 0.818. Incorporating the LLM as either an additional or substitute evaluator did not significantly alter the reliability of the underlying evaluation instrument [18]. Similarly, Grolleau et al. (2025) reported that an LLM jury evaluating factual clinical summaries achieved a Cohen’s κ of approximately 0.81 against a physician-panel reference standard, with performance statistically noninferior to that of a single human evaluator [19]. Combined with our findings, these studies support that automated evaluators can be effective when the task is bounded, the evaluation rubric is explicit, and relevant clinical evidence is available to the judge [1].

However, the literature also demonstrates that this performance should not be generalized across all forms of clinical evaluation. Williams et al. (2026) compared multiple LLM judges with clinicians evaluating responses to real-world questions from community health workers and found that the highest-performing judge achieved human-equivalent evaluation on only four of 11 criteria, with performance varying by model, evaluation dimension, and language [20]. A multicenter study involving more than 400 physicians also found that, while automated evaluators were directionally aligned with physician assessments, they did not fully reproduce the contextual and clinical nuances of human judgment [21]. Collectively, these findings indicate that LLM-as-a-Judge performance is not universal but depends on the specific context and evaluation task in which it is deployed, and cannot be attributed solely to the intrinsic properties of a particular model.

The findings also align with broader principles of AI validation across biomedical applications. In multi-class skin-lesion classification, Iqbal et al. (2021) evaluated performance across heterogeneous lesion categories and multiple datasets using complementary metrics and class-specific analyses, illustrating that aggregate accuracy alone may obscure important differences in performance across clinically relevant classes [22]. Similarly, Iqbal et al. (2025) evaluated an automated framework for identifying stem-cell-derived endothelial cells using independently partitioned datasets, domain-expert reference labels, and multiple complementary performance measures [23]. Although these studies address biomedical imaging rather than LLM evaluation, they reinforce the importance of task-specific validation, class-aware performance assessment, and comparison against clinically or biologically meaningful reference standards.

Our results may therefore be partly explained by the design of the evaluation framework. Rather than asking the LLM to provide an unconstrained judgment of response quality, the judge followed the same prespecified framework used for physician review, separating scope determination from response appropriateness and mapping these decisions to TP, FN, TN, or FP categories. The judge was additionally grounded in the clinical evidence available for each interaction. This combination of explicit criteria, task decomposition, few-shot examples, and evidence grounding may reduce some of the ambiguity or hallucination associated with open-ended LLM evaluation [1,6].

Consistent with the four-class findings, the secondary binary correctness analysis further demonstrated high concordance between the LLM Judge and physician evaluation. The slightly higher agreement observed in the binary analysis compared with the four-class analysis likely reflects the reduced complexity of distinguishing Correct from Incorrect responses after collapsing the original TP, TN, FP, and FN categories. The higher sensitivity and PPV relative to specificity and NPV also indicate stronger agreement for responses considered Correct, although these differences should be interpreted in the context of the greater prevalence of Correct responses in the validation cohort. The absence of significant asymmetry by McNemar’s test further suggests that discordant classifications were not systematically biased toward either Correct or Incorrect determinations.

From an operational perspective, these findings support the use of LLM Judges as assistive tools for scaling the evaluation of patient-facing AI systems. An automated Judge could perform initial screening of large volumes of interactions and prioritize uncertain, discordant, or potentially unsafe responses for physician review. Such an approach could reduce the burden of continuous human monitoring [24,25] and shift physician effort from exhaustive review toward adjudication of complex or safety-sensitive cases.

Compared with physician-only review, an LLM Judge offers potential advantages in scalability, rapid evaluation of large interaction volumes, and consistent application of prespecified evaluation criteria [1,18,19]. Structured LLM-based evaluation can also incorporate free-text clinical context and supporting evidence when assessing response quality, potentially facilitating standardized screening and prioritization of cases for human review. These advantages are balanced by important limitations: performance may depend on the underlying model, prompt design, evaluation criteria, and inference configuration, and LLM judges may exhibit biases including self-preference and sensitivity to how evaluation tasks are formulated [6]. Moreover, performance may be less reliable for subjective, contextual, or safety-sensitive clinical judgments than for more objective criteria. Automated evaluation may also introduce correlated errors or automation bias when one AI system evaluates another [20,26].

Importantly, these potential advantages should not be interpreted as demonstrating superiority of automated evaluation or establishing formal statistical equivalence with physicians. Rather, our findings demonstrate that, for the specific evaluation task and framework studied, the LLM Judge produced classifications and aggregate performance estimates that closely approximated those obtained through physician evaluation [27]. These findings support a complementary role for automated evaluation within human-in-the-loop monitoring workflows, rather than a replacement of expert clinical oversight.

(b) Ethical Considerations

The use of an LLM to evaluate another AI system introduces an additional layer of automation into an already AI-mediated clinical communication process. Although this may improve scalability, it also creates the possibility that errors produced by one automated system may be overlooked, reinforced, or incorrectly validated by another. In healthcare, minor inaccuracies related to medications, postoperative symptoms, wound care, or emergency escalation may have consequences that are not adequately represented by linguistic quality alone. In our study, however, some discordant cases reflected more conservative evaluation by the LLM Judge, with physician-accepted responses classified as incorrect because the Judge applied stricter criteria for response specificity, contextual completeness, or escalation guidance.

For this reason, our findings support a human-in-the-loop model rather than autonomous replacement of physician evaluation. Recent reviews of LLM-as-a-judge approaches in healthcare similarly conclude that automated judges are best positioned as supportive rather than substitutive tools, particularly when clinical judgments are subjective, ambiguous, or safety-sensitive [1,26,28]. A recent large-scale comparison of physician and AI evaluators reached a similar conclusion, suggesting that automated evaluation may be useful for triage or prescreening but cannot fully substitute for physician-centered assessment [21].

This approach is consistent with broader ethical and regulatory principles governing AI in healthcare. The World Health Organization has emphasized transparency, accountability, protection of human autonomy, expert supervision, rigorous evaluation, and appropriate governance in the development and deployment of generative AI for health [29]. Similarly, Article 14 of the EU AI Act requires effective human oversight for high-risk AI systems, including the ability of human operators to understand system limitations, recognize automation bias, monitor system behavior, and disregard or override automated outputs when necessary [30]. U.S. FDA and international Good Machine Learning Practice principles likewise emphasize evaluation of the performance of the human–AI team and lifecycle approaches to maintaining safety and effectiveness [31].

The regulatory applicability of these frameworks depends on the intended use and classification of a specific AI system; nevertheless, the principles are directly relevant to governance of automated clinical evaluation. An LLM judge should therefore not be treated as an independent source of clinical authority. Its role is better conceptualized as a quality-assurance mechanism operating under defined rules, with transparent outputs, auditable evidence, and predefined thresholds for escalation to human reviewers. Particular consideration should be given to avoiding automation bias, whereby reviewers may defer excessively to an apparently confident automated classification despite contradictory clinical evidence.

(c) Study Limitations

This study has several limitations. First, although the study included patients from two Mayo Clinic sites (Florida and Rochester) and multiple surgical specialties, it was conducted within a single healthcare system and evaluated one patient-facing AI application. The inclusion of multiple sites and surgical specialties increased the heterogeneity of the validation cohort; however, the findings may not generalize to other healthcare systems, patient populations, or conversational AI platforms. Prior studies have similarly demonstrated that LLM-judge performance varies by model, task, evaluation criterion, and clinical context [20,21].

Second, the judge was implemented using a specific LLM, prompt, and evidence-grounding framework. Model updates or alternative configurations may yield different results, and model-family familiarity between AIVA and the LLM Judge may also have contributed to the present findings. They should therefore be interpreted as validation of the complete evaluation framework rather than of LLM judges broadly [20]. Additionally, although the LLM Judge used its own knowledge base to support evaluation and reasoning, overlap between the clinical evidence available to the two systems may still contribute to correlated errors. The present study evaluated end-to-end Judge performance and did not separately distinguish errors attributable to retrieval, knowledge-base limitations, response generation, or Judge classification.

Third, physician-generated questions were intentionally used during prompt development to probe predefined edge cases but may not fully represent the linguistic variability and ambiguity of real patient questions. This limitation was mitigated by restricting simulated interactions to prompt refinement and conducting validation using real patient–AIVA interactions.

In addition, the validation cohort was imbalanced across classification categories, with relatively few FP (n = 33) and TN (n = 44) interactions. Consequently, category-specific agreement estimates for these less frequent classes were less precise, as reflected by their wider confidence intervals, and should be interpreted cautiously. This limits strong conclusions regarding Judge performance in these categories despite the observed agreement estimates.

Finally, the individual patient–AIVA interaction was the primary unit of analysis, and some patients contributed multiple interactions. Interactions originating from the same patient may share procedural context, clinical concerns, language characteristics, or retrieved evidence and therefore may not be fully independent. The present analysis did not explicitly account for within-patient clustering; consequently, CIs may underestimate uncertainty in the presence of meaningful intrapatient correlation. The reported metrics should therefore be interpreted as interaction-level estimates.

(d) Future Directions

Future studies should validate this framework across other institutions, patient populations, conversational AI systems, and assess classification stability across repeated runs, alternative Judge models, and controlled variations in prompt formulation and few-shot examples. Particular attention should be given to uncommon but clinically important failure modes, including medication errors, postoperative complications, inappropriate emergency escalation, and situations in which available clinical evidence is incomplete or conflicting. Patient-level analyses should evaluate performance heterogeneity across patients and the potential influence of patients contributing larger numbers of interactions.

Prospective studies should also evaluate risk-stratified human–AI workflows in which high-confidence, low-risk interactions undergo automated screening while uncertain, discordant, or safety-sensitive cases are escalated for physician review [32]. Such approaches may preserve human oversight while reducing monitoring burden. Future work should quantify effects on physician workload, evaluation time, cost, and patient safety, as well as assess performance stability following model updates [1,20].

Finally, studies designed to formally establish equivalence or noninferiority to physician evaluation should prespecify clinically acceptable margins and use appropriately powered statistical designs. Until then, LLM judges should be considered assistive evaluation tools rather than replacements for expert clinical oversight.

## 5. Conclusions

This study demonstrates that a structured, clinically grounded LLM-as-a-Judge framework can closely reproduce physician evaluation of AI-generated responses to patients’ periprocedural questions. The high agreement with physician classifications and similar estimates of AIVA performance support the potential use of LLM judges as scalable, assistive tools for monitoring patient-facing AI systems. However, these findings should not be interpreted as establishing equivalence to or replacement of physician evaluation. LLM-based evaluation may complement clinical oversight by facilitating large-scale screening and prioritizing uncertain or safety-sensitive interactions for human review. Further prospective and external validation is needed to determine its generalizability, operational impact, and role within human–AI oversight frameworks.

## Figures and Tables

**Figure 1:**
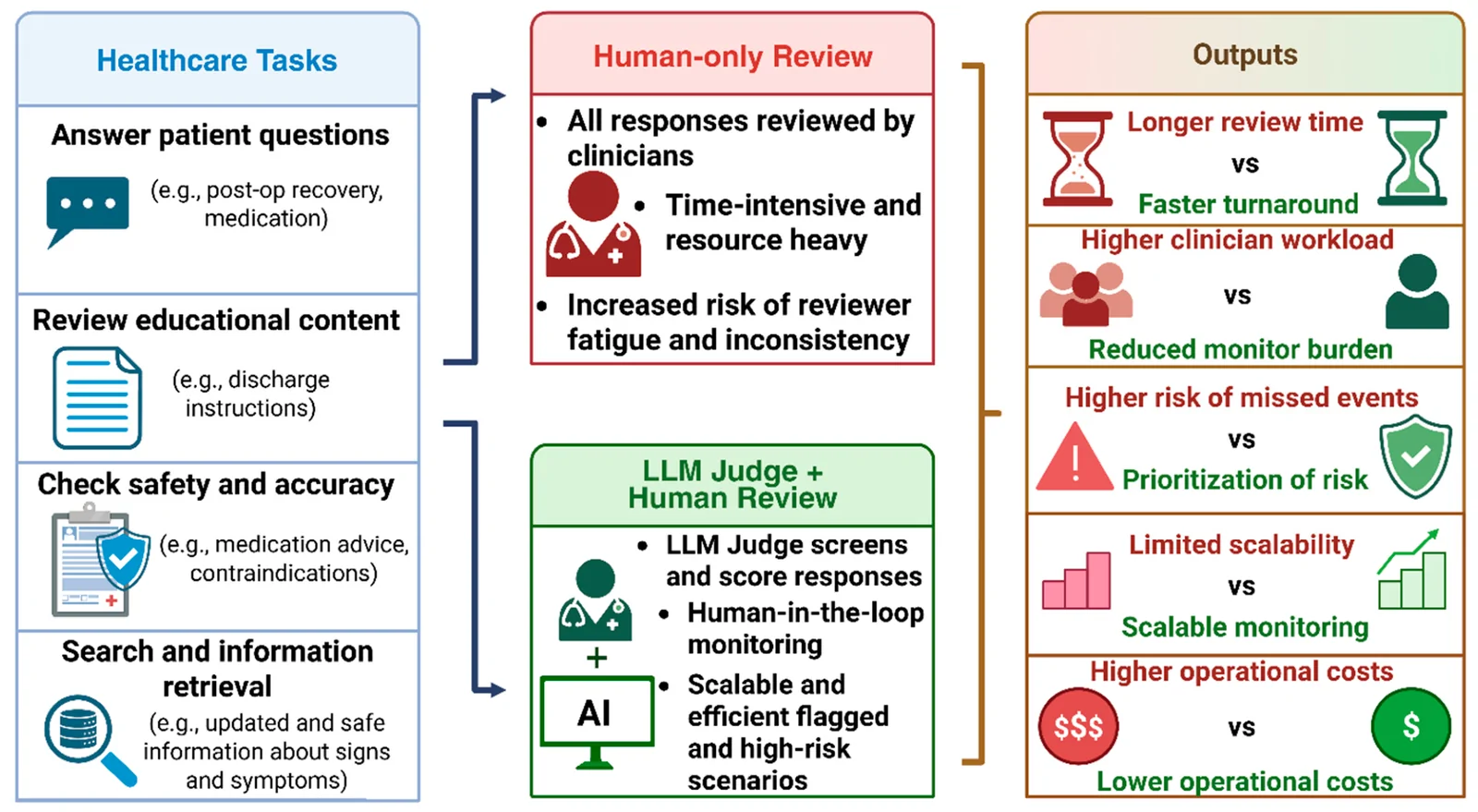
Conceptual framework for LLM-assisted evaluation of healthcare AI tasks, illustrating how an LLM Judge may reduce human review burden while preserving clinician oversight. Created in BioRender. Collaco, B. (2026) https://BioRender.com/cdbce9y, accessed on 23 August 2026.

**Figure 2:**
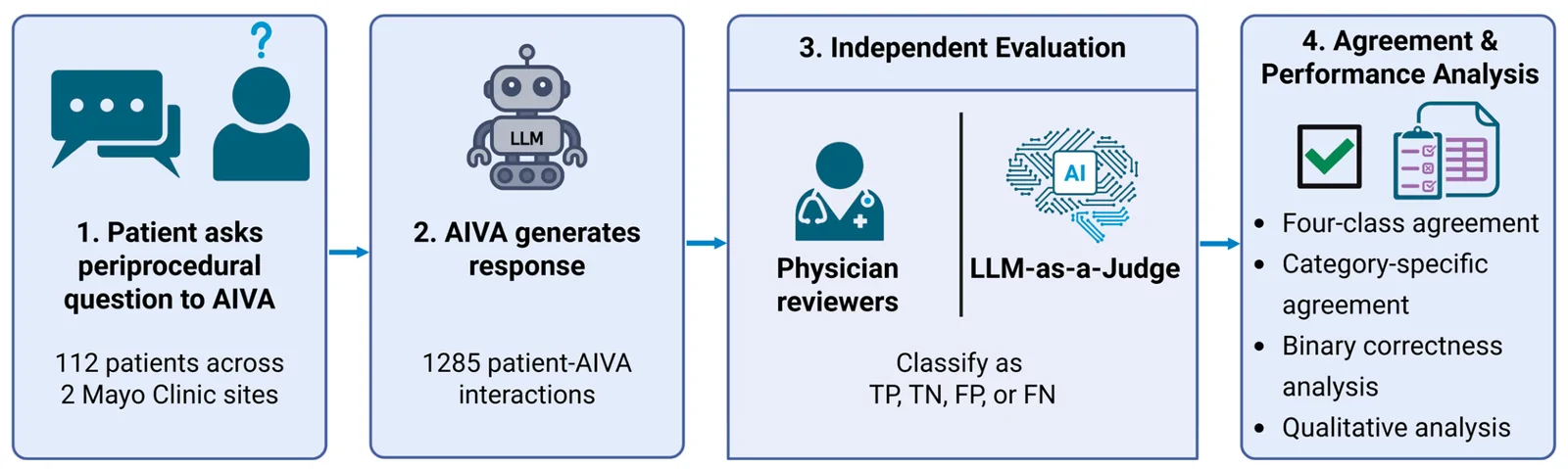
Overview of the study design and validation workflow. Created in BioRender. Collaco, B. (2026) https://BioRender.com/cdbce9y, accessed on 23 August 2026.

**Figure 3:**
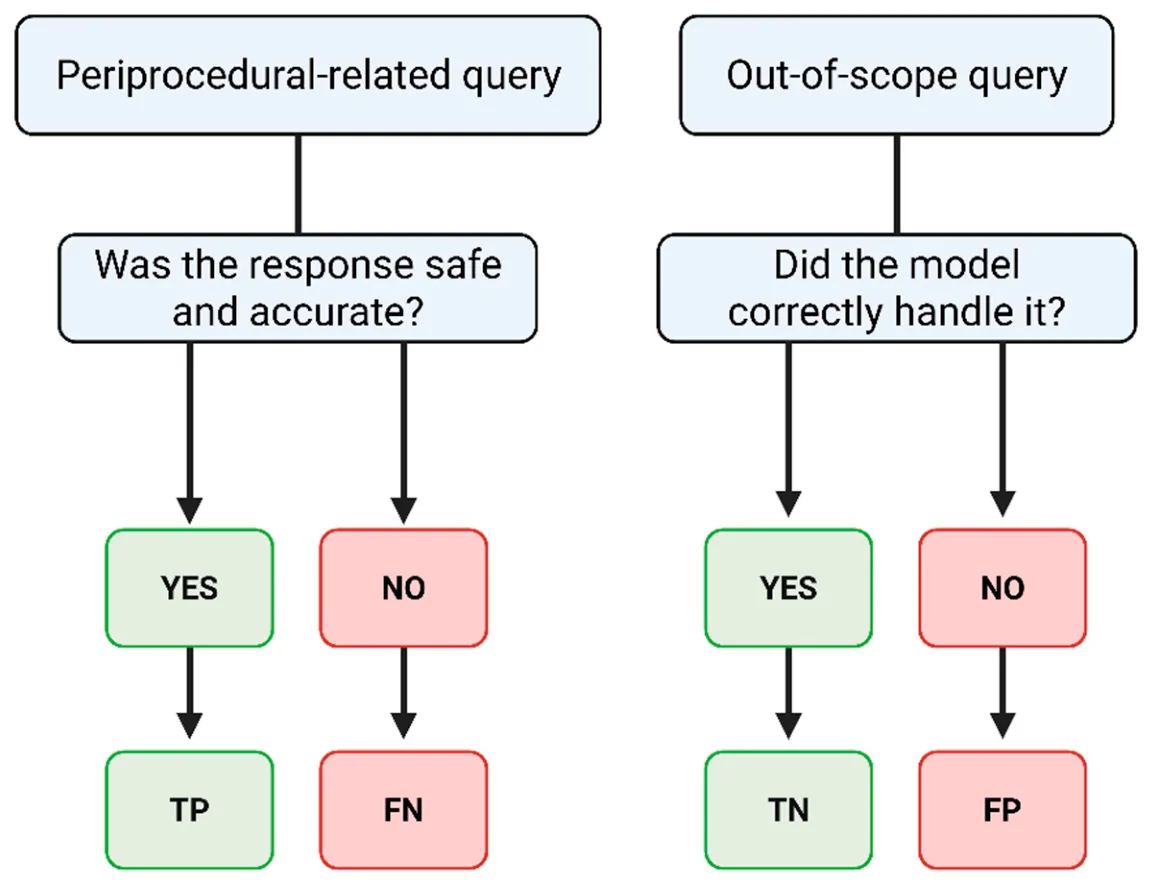
Classification framework used to categorize AIVA responses. Created in BioRender. Collaco, B. (2026) https://BioRender.com/cdbce9y, accessed on 23 August 2026.

**Figure 4:**
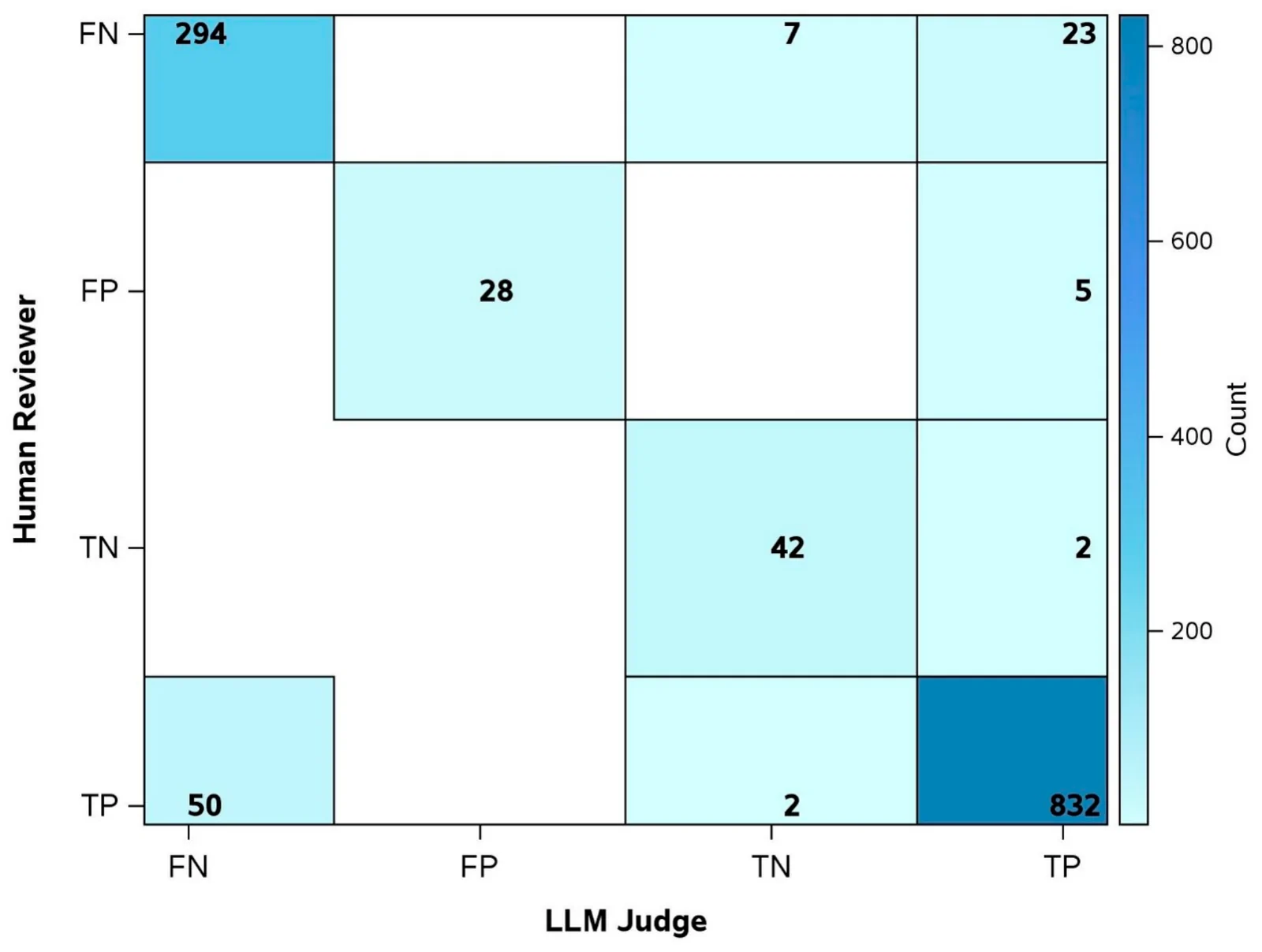
Heatmap of Agreement between the Human Reviewer and LLM Judge across the Four Original Classification Categories. The heatmap demonstrates a strong concentration of classifications along the diagonal, visually reflecting the high concordance between physician and LLM Judge assessments across the four categories. The most prominent off-diagonal disagreement involved physician-classified TP interactions assigned as FN by the LLM Judge (*n* = 50), followed by physician-classified FN interactions assigned as TP (*n* = 23), while other cross-category disagreements were infrequent.

**Table 1:** Distribution of Physician and LLM-as-a-Judge Classifications of Patient–AIVA Interactions. Values are numbers of interactions. TP, true positive; FN, false negative; TN, true negative; FP, false positive.

Category	Human Evaluation (*n*)	LLM-as-a-Judge (*n*)
FN	324	344
FP	33	28
TN	44	51
TP	884	862
Total Interactions	1285	

**Table 2:** AIVA Performance Metrics Derived From Physician Evaluation and LLM-as-a-Judge Classifications. Values are presented as estimates with 95% CIs. *PPV, positive predictive value; NPV, negative predictive value; CI, confidence interval.*

Metric	Human Evaluation	LLM-as-a-Judge
Overall Accuracy, % (95% CI)	72.2 (69.7, 74.6)	71.1 (68.5, 73.5)
Sensitivity (Recall), % (95% CI)	73.2 (70.6, 75.6)	71.5 (68.9, 74.0)
Specificity, % (95% CI)	57.1 (46.0, 67.6)	64.6 (53.6, 74.2)
PPV (Precision), % (95% CI)	96.4 (95.0, 97.4)	96.9 (95.5, 97.8)
NPV, % (95% CI)	12.0 (9.0, 15.7)	12.9 (10.0, 16.6)
F1-Score (95% CI)	0.832 (0.811, 0.851)	0.823 (0.801, 0.843)

**Table 3:** Four-Class Agreement Matrix Between Human Reviewer and LLM-as-a-Judge.

		LLM-as-a-Judge, *n* (%)				Total
		FN	FP	TN	TP	
**Human Reviewer** **(Reference Standard)**	FN	294 (90.7%)	0 (0%)	7 (2.2%)	23 (7.1%)	324
	FP	0 (0%)	28 (84.9%)	0 (0%)	5 (15.1%)	33
	TN	0 (0%)	0 (0%)	42 (95.5%)	2 (4.5%)	44
	TP	50 (5.7%)	0 (0%)	2 (0.2%)	832 (94.1%)	884
	Total	344	28	51	862	1285
**Overall agreement, % (95% CI)**	93.1 (91.7, 94.5)					
**Cohen’s kappa (95% CI)**	0.852 (0.823, 0.882)					

Note: AI-generated responses were classified into four categories (TP, TN, FP, and FN) by the human reviewers and the LLM Judge. The table presents the cross-classification of categories assigned by the LLM Judge against those assigned by the human reviewers. Overall agreement was calculated as the proportion of responses for which both evaluations assigned the same category. Cohen’s kappa (κ) was used to quantify inter-rater agreement beyond that expected by chance. The 95% CIs were calculated using the binomial method for overall agreement and the standard error method for Cohen’s kappa.

**Table 4:** Category-Specific Agreement Between Human Reviewer and LLM-as-a-Judge.

Category	Human Reviewer (*n*)	LLM-as-a-Judge (*n*)	Agreement (95% CI)
FN	324	294	90.7% (87.6%, 93.9%)
FP	33	28	84.9% (72.6%, 97.1%)
TN	44	42	95.5% (89.3%, 100%)
TP	884	832	94.1% (92.6%, 95.7%)

Note: Category-specific agreement was calculated as the proportion of responses classified into the specified category by the human reviewer that were also classified into the same category by the LLM-as-a-Judge. 95% CIs for category-specific agreement were calculated using the binomial method.

**Table 5:** Binary Classification of AI-Generated Response Correctness by the LLM-as-a-Judge Compared with the Human Reviewer.

LLM-as-a-Judge	Reviewer (Reference Standard)		Total
	Correct	Incorrect	
**Correct**	878	35	913
**Incorrect**	50	322	372
**Total**	928	357	1285
**Cohen’s kappa**	0.837 (0.804, 0.871)		
**McNemar’s Test**	0.11		
**Accuracy (95% CI)**	93.4 (92.0, 94.7)		
**Sensitivity/Recall (95% CI)**	94.6 (93.2, 96.1)		
**Specificity (95% CI)**	90.2 (87.1, 93.3)		
**PPV/Precision (95% CI)**	96.2 (94.9, 97.4)		
**NPV (95% CI)**	86.6 (83.1, 90.0)		
**F1 score (95% CI)**	0.954 (0.944, 0.964)		

Note: Correct responses comprised original TP or TN classifications; Incorrect responses comprised FP or FN classifications. PPV, positive predictive value; NPV, negative predictive value; CI, confidence interval.

**Table 6:** Qualitative analysis between Physician-TP and LLM Judge-FN Discordant Interactions.

Primary Discordance Theme	*n* (%)	What the Judge Was Reacting to
**Incomplete or insufficiently specific response**	25 (50)	The response was generally relevant but did not directly answer the precise question, such as timing, appearance, technique, prognosis, or rationale.
**Generic or overly conservative emergency escalation**	10 (20)	AIVA gave a generic 911/emergency response rather than addressing the postoperative concern or directing the patient to the care team.
**Context/evidence mismatch or inappropriate guideline application**	7 (14)	The Judge identified evidence that did not adequately match the procedure, demographic, clinical context, or treatment being discussed.
**Temporal ambiguity or conflicting recovery guidance**	5 (10)	The Judge interpreted the response as inconsistent with the patient’s postoperative timing or as giving conflicting timeline information.
**Missing safety caveat or insufficient escalation guidance**	3 (6)	The response was largely appropriate, but the Judge considered an important safety precaution, warning sign, or escalation recommendation to be missing.

## Data Availability

The data supporting the findings of this study are available within the article and its Supplementary Materials. Additional information may be available from the corresponding author upon reasonable request.

## Supplementary Materials

The following supporting information can be downloaded at https://www.mdpi.com/article/10.3390/bioengineering13101115/s1. Supplementary File S1: Patient–AIVA Interactions and LLM Judge and Physician Classifications; Supplementary File S2: Pre-Adjudication Physician Reviewer Classifications and Final Consensus Labels; Supplementary File S3: Pre-adjudication interrater agreement between physician reviewers; Supplementary File S4: LLM Judge Validation Cases and Classification Results; Supplementary File S5: LLM Judge Prompt and Classification Framework; Supplementary File S6: Discordant LLM Judge and Physician Classifications and Error Categories.

## Abbreviations

AI	Artificial Intelligence
AIVA	Artificial Intelligence Virtual Assistant
CI	Confidence Interval
FN	False Negative
FP	False Positive
HEC	Health Education Content
HIL	Health Information Library
IRB	Institutional Review Board
LLM	Large Language Model
NPV	Negative Predictive Value
OOS	Out of Scope
PEL	Patient Education Library
PPV	Positive Predictive Value
TN	True Negative
TP	True Positive
