# Supplementary material for: Development and Clinical Validation of an Automated LLM Judge for Evaluating Perioperative Patient Questions
Source: Bioengineering (Basel). 2026 Sep 24;13(10):1115. doi: 10.3390/bioengineering13101115 (PMC13701884; doi:10.3390/bioengineering13101115)
Supplement: Supplementary file 1 [file bioengineering-13-01115-s001.zip › Supplementary File S3.pdf]

		Reviewer 2				Total
		FN	FP	TN	TP	
**Reviewer 1**	FN	312	0	5	17	334
	FP	0	31	0	2	33
	TN	2	0	41	0	43
	TP	17	4	1	853	875
	**Total**	331	35	47	872	1285
**Overall agreement, %**	**96.3**					
**Cohen's kappa (95% CI)**	**0.920 (0.898, 0.942)**					

Supplementary File S3. Pre-adjudication interrater agreement between physician reviewers.
